# Supplementary material for: The Influence of Role Models on the Sedentary Behaviour Patterns of Primary School-Aged Children and Associations with Psychosocial Aspects of Health
Source: Int J Environ Res Public Health. 2020 Jul 24;17(15):5345. doi: 10.3390/ijerph17155345 (PMC7432808; doi:10.3390/ijerph17155345)
Supplement: Supplementary file 1 [file ijerph-17-05345-s001.pdf]

Table S1: Sociodemographic of children.

	All	Boys	Girls
	n = 83	*n* = 35	*n* = 48
**Racial background**			
White	99%	96%	100%
Indian	1%	4%	0%
**Location**			
Donegal (Ireland)	51%	50%	51%
Londonderry (UK)	49%	50%	49%
**Family unit**			
2 parents	82%	82%	81%
1 parent	17%	18%	16%
other	1%	0%	2%
**Father's Education**			
Primary	9%	0%	14%
GCSE or Junior Cert*	31%	39%	26%
A-Level or Leaving Cert*	20%	21%	19%
HND or Certificate*	10%	11%	9%
Degree	14%	14%	14%
Postgraduate	15%	14%	16%
N/A	2%	0%	2%
**Mother's Education**			
Primary	6%	7%	5%
GCSE or Junior Cert*	24%	18%	29%
A-Level or Leaving Cert*	14%	14%	14%
HND or Certificate*	13%	18%	10%
Degree	20%	32%	12%
Postgraduate	20%	11%	26%
N/A	2%	0%	5%
**Work Status of Father**			
Employed full-time	67%	59%	71%
Employed part-time	9%	7%	10%
Student	1%	4%	0%
At home with children	4%	7%	2%
Unemployed	7%	4%	10%
Not applicable	3%	7%	0%
Other	6%	11%	2%
Prefer not to answer	3%	0%	5%
**Work Status of Mother**			
Employed full-time	32%	41%	26%
Employed part-time	25%	22%	26%
Student	4%	4%	5%
At home with children	25%	19%	29%
Unemployed	6%	4%	7%
Not applicable	4%	4%	5%
Other	3%	7%	0%
Prefer not to answer	1%	0%	2%

Table S2. Scores for psychosocial variables by gender and BMI category

Variable	Rosenberg Self-Esteem Scale	Strengths & Difficulties Questionnaire
Gender (all)	16.6 (2.0)	17.5 (4.7)
Boys	16.9 (1.9)	17.9 (5.4)
Girls	16.4 (2.2)	17.3 (4.1)
BMI category		
Thin	17.3 (2.2)	14.1 (3.3)
Average	16.9 (2.0)	16.7 (4.9)
Overweight	16.4 (2.3)	19.2 (4.8)
Obese	16.2 (1.3)	19.4 (4.1)

Values are means (SD).

Table S3. Proportion of sample scoring in each psychosocial category**.**

Variable	All (%)	Boys (%)	Girls (%)
**RSES**			
Low (<15)	23.0	24.2	5.9
Normal (15-25)	44.6	45.5	94.1
High (26-30)	32.4	30.3	0
**SDQ**			
Normal (low)	23.0	24.2	22.0
Borderline	44.6	45.5	43.9
Abnormal (high)	32.4	30.3	34.1

Values are means (SD) or percentages. RSES = Rosenberg Self-Esteem Scale; SDQ = Strengths and Difficulties Questionnaire
